# 

**Published:** 1971-07

**Authors:** 


The
Bristol Medico-Chirurgical Journal
(Incorporating the Medical Journal of the South-West)
Established 1883

Vol. 86 (iii) JULY 1971 No. 319

### BRISTOL MEDICO-CHIRURGICAL JOURNAL

Editor: Dr. N. J. Brown, M.B., F.R.C.P., F.R.C.Path.

Published Quarterly

Annual Subscription 80p (16/-) Post Free

### BRISTOL MEDICO-CHIRURGICAL SOCIETY

**President 1970-71 : Dr. Beryl Corner**

**President-elect : Dr. C. C. Morgans**

**Honorary Secretary : Dr. I. S. Bailey, 7 Percival Road Bristol 8.**

**Honorary Treasurer : Dr. Frank Ross, Bristol Royal Infirmary, Bristol 2.**

The Society was founded in 1874. In 1883 publication of the Journal began and has continued quarterly ever since then. During the years 1949 to 1962 it appeared under the title of "The Medical Journal of the South West".

### THE BRISTOL MEDICO-CHIRURGICAL JOURNAL

### Editorial Committee

Editor — Dr. N. J. Brown, Southmead Hospital, Bristol.

Members — Mr. J. B. M. Roberts.
Dr. M. B. Lennard.
Professor R. C. Wofinden.
Dr. D. W. Wright.
The Hon. Treasurer and Hon. Secretary of the Society.

Business Manager — Dr. P. J. F. Baskett, Frenchay Hospital, Bristol.

Secretary — Mr. B. P. Jones, The Library, Medical School, University Walk, Bristol BS8 1TD.

### NOTICE TO CONTRIBUTORS

The Journal is especially concerned to provide a place for the recording of medical thought, interests, and practice in and around Bristol. It therefore welcomes articles that, as well as being of immediate interest to members, will document the local and contemporary medical scene for our successors.

Original articles are invited provided that they have not been published elsewhere. References in the text should be by author's name, with year of publication in parentheses; they should be listed at the end in alphabetical order, the name of each journal or book being given in full — not abbreviated — and the title of the article added. Illustrations should be supplied ready for reproduction. Line drawings should be in Indian ink on firm white paper or board. The author will be informed if it is found necessary to ask him to pay for the making of blocks.

The following contributions will be especially welcome : notes of forthcoming events, meetings held, degrees conferred, staff appointments, honours and public appointments and other local news.

Twenty-five reprints of his article will be supplied free to each contributor. Quotations for larger quantities will be sent out with proofs and must be ordered when the corrected proofs are returned.

### Society Report

**Bristol Medico-Chirurgical Society**

The sixth meeting of the session was held on 10th March when Professor O. H. Wolff of the Institute of Child Health, London, addressed the Society on "Priorities in Paediatrics".

At this meeting it was decided that the subscription should be £4 per annum, that the subscription to the Journal for non-members of the Society should be £2 per annum and that the Society should seek Registration under the Charities Act. The necessary alteration to the Rules were agreed. The revised rules will be published in the October number of the Journal.

At the seventh meeting on 14th April there was a Symposium on the Problems of Drug Addiction arranged by Dr. A. M. G. Campbell. The principal speakers were Dr. R. de Alancon of the M.R.C. Clinical Psychiatry Unit, Chichester, and Prof. D. M. Paton, Professor of Pharmacology, Oxford University.

The final meeting of the session on 12th May was addressed on the subject of "The General Medical Council" by the Council's President, the Rt. Hon. Lord Cohen of Birkenhead.

### The Medical Library

In 1890 the Bristol Medico-Chirurgical Society founded a medical library which in 1892 was combined with that of the Bristol Medical School. This became the University of Bristol Medical Library in 1925 and an agreement in that year confirmed the privilege of Members of the Society to use the University Medical Library.

### A Selection of Recent Additions to the Medical Library

ABC OF DRUG ADDICTION. (Wright). Presented by John Wright & Sons Ltd.

AMBROSE, E. J. and EASTY, D. M.: Cell biology. (Nelson).

ANDREWES, Sir C.: Viruses and cancer. (Weidenfeld & Nicolson).

ARCOS, J. C. et al: Chemical induction of cancer. Vol. 1. (Academic Press).

ARMITAGE, P.: Statistical methods in medical research. (Blackwell).

BABEL, J. et al.: Ultrastructure of the peripheral nervous system and sense organs. (Thieme).

BARBER, H. R. K. and GRABER, E. A. eds.: Gynecological oncology. (Excerpta Medica).

BARRY, R. D. and MAHY, B. W. J.: Biology of large RNA viruses. (Academic Press).

BASERGA, R. and MALAMUD, D.: Autoradiography: techniques and application. (Harper & Row).

BERG, J. M. et al.: The De Lange syndrome. (Pergamon Press).

BERGSTRÖM, S. and SAMUELSSON, B. eds.: Prostaglandins: proc. of the 2nd Nobel Symposium. (Interscience).

BJÖRKMAN, N.: Atlas of placental fine structure. Baillière).

BOYD, W.: Textbook of pathology. Ed. 8. (Kimpton). Presented in memory of Paul Esser by his friends on the firm.

BOYLE, J. A. and BUCHANAN, W. W.: Clinical rheumatology. (Blackwell).

BRITISH CANCER COUNCIL: Two meetings: 'People and cancer'; 'The problem of relevance in cancer'. (British Cancer Council).

BROWN, H.: Hepatic failure. (Thomas).

BURKE, J. D.: Cell biology. (Williams & Wilkins).

BURNS, L. E. and WORSLEY, J. L. eds.: Behaviour therapy in the 1970s. (Wright). Presented by John Wright & Sons Ltd.

CAMPBELL, E. J. M. et al.: The respiratory muscles: mechanics and neural control. (Lloyd-Luke).

CAMPS, F. E.: Practical forensic medicine. Ed. 2. (Hutchinson).

CAPLAN, G.: Theory and practice of mental health consultation. (Tavistock).

CIBA FOUNDATION: Hormones and the immune response. (Churchill).

CIBA FOUNDATION: Law and ethics of transplantation. (Churchill).

CIBA FOUNDATION: Molecular properties of drug receptors. (Churchill).

CLARKE, C. A.: Human genetics and medicine. (Arnold).

COELIAC HANDBOOK 1970. (Coeliac Society).

CUTHBERT, A. W.: Calcium and cellular function. (Macmillan).

D'ABREU, A. L. et al.: Intrathoracic crises. (Butterworth).

DANIEL, W. A.: The adolescent patient. (Mosby). Presented by Dr. J. Apley.

DAWSON, R. M. C. et al., eds.: Data for biochemical research. Ed. 2. (Oxford Univ. Press).

EHRENPREIS, S. and SOLNITZKY, O. C. eds.: Neurosciences research. Vol. 1. (Academic Press).

ELLENBERG, M. and RIFKIN, H.: Diabetes mellitus. Ed. 2. (McGraw-Hill).

FLETCHER, G. H. and JING, B.: Atlas of tumor radiology Vol. 1: Head and neck. (Year Book).

FLINT, T. and CAIN, H. D.: Emergency treatment and management. Ed. 49. (Saunders).

FLOCKS, R. H. and CULP, D.: Surgical urology. Ed. 3. (Year Book).

FRANCIS-WILLIAMS, J.: Children with specific learning difficulties. (Pergamon Press).

FRANCOIS, J.: Congenital cataracts. (Royal Van-Gorcum).

FRY, R. J. M., et al., eds.: Late effects of radiation. (Taylor & Francis).

GASTON, P. J.: The care, handling and disposal of dangerous chemicals (Northern Publishers (Aberdeen) Ltd.).

GODBER, Sir G.: Medical care (Ciba 20th Anniversary Lecture). (Churchill).

GRAVES, F. T.: The arterial anatomy of the kidney: the basis of surgical technique. (Wright). Presented by John Wright & Sons Ltd.

GREENLAND, C.: Mental illness and civil liberty. (Bell).

HAFEZ, E. S. E.: Reproduction and breeding techniques for laboratory animals (Baillière).

HANDBOOK OF SENSORY PHYSIOLOGY: Vol. 1: Principles of receptory physiology, ed. by W. R. Loewenstein. (Springer).

HARE, E. H. and WING, J. K. eds.: Psychiatric epidemiology: proc. internat. symp. Aberdeen 1969. (Oxford Univ. Press).

HAWKER, L. E. and LINTON, A. H. eds.: Micro-organisms: function, form and environment. (Arnold). Presented by the Editors.

HEWETT, S. et al.: The family and the handicapped child: a study of cerebral palsied children in their homes. (Allen & Unwin). Presented by Dr. J. Apley.

HOLDSWORTH, W. G.: Cleft lip and palate. Ed. 4. (Heinemann).

HOPTON, D. S. and DAVIES, I. J. T.: Practical hints for housemen. (Lloyd-Luke).

HORROBIN, D. F.: Principles of biological control. (Med. Tech. Publ.).

HURST, J. W. and LOGUE, R. B.: The heart. Ed. 2. (McGraw-Hill).

JAMIESON, G. A. and GREENWALT, T. J.: Red cell membrane: structure and function. (Lippincott).

JONES, R. H.: The doctor and the social services (Heath Clark Lecture 1969). (Athlone Press).

KING, B. H.: Perceptions of illness and medical practice. (Russell Sage Foundation).

KNUTSON, A. L.: The indivdual, society and health behaviour. (Russell Sage Foundation).

LLEWELLYN-JONES, D.: Fundamentals of obstetrics and gynaecology. Vol. 2: Gynaecology. (Faber).

M. D. ANDERSON HOSPITAL AND TUMOR INSTITUTE: Neoplasia in childhood. (Year Book).

MARRIOTT, F. H. C.: Basic mathematics for the biological and social sciences. (Pergamon).

MASTERS, W. and JOHNSON, V. E.: Human sexual inadequacy. (Churchill).

MATHÉ, G. ed.: Advances in the treatment of acute (blastic) leukaemias. (Recent results in cancer research, 30). (Heinemann).

MATHÉ, G.: Therapeutic strategy in acute lukaemia. (Queen Anne Pr. for Leukaemia Research Fund).

MAURO, A. et al., eds.: Regeneration of striated muscle and myogenesis. (Excerpta Medica).

MEDICAL RESEARCH COUNCIL—COMMITTEE ON BIOCHEMICAL RESEARCH IN PSYCHIATRY: Biochemical research in psychiatry: survey and proposals (D. A. K. Black). (HMSO).

MEEK, G. A.: Practical electron microscopy for biologists. (Wiley).

MEITES, J.: Hypophysiotropic hormones of the hypothalamus. (Livingstone).

MILHORAT, A. T. ed.: Exploratory concepts in muscular dystrophy and related disorders. (Excerpta Medica).

MILLER, A. L. and LEHMAN, R. H.: Practical guide on hearing-impaired children. (Thomas).

MONIF, G. R. G.: Viral infections of the human fetus. (Collier-Macmillan).

MORISON, J. E.: Foetal and neonatal pathology. Ed. 3. (Butterworth).

MORLEY, M. E.: The development and disorders of speech in childhood. Ed. 2. (Livingstone). Presented by Dr. J. Apley.

MOROWITZ, H. J.: Entropy for biologists. (Academic Press).

NEALE, A. V.: The British Paediatric Association, 1952-1968. (Pitman).

NEWBURGH, L. H.: Physiology of heat regulation and the science of clothing. (Hafner).

PAGE, I. H.: Serotonin. (Year Book).

PEACOCK, E. E. and VAN WINKLE, W.: Surgery and biology of wound repair. (Saunders).

PENN, I.: Malignant tumors in organ transplant recipients (Recent results in cancer research, 35) (Springer).

ROSEMBERG, E. and PAULSEN, C. A. ed.: The human testis (Advances exper.med.biol., 10). (Plenum).

ROYAL SOCIETY OF HEALTH: Health education audio-visual aids. (Royal Society of Health).

RUTTER, M. L. et al., eds.: Education, health and behaviour. (Longman).

SCURR, C. and FELDMAN, S. eds.: Scientific foundations of anaesthesia. (Heinemann).

SELLS, S. B. ed.: Definition and measurement of mental health. (U.S. Govt. Printing Office).

SHAPTON, D. A. and BOARD, R. G.: Isolation of anaerobes (Soc. for Applied Bacteriology, Technical series, 5). (Academic Press).

SILVER, I. H. ed.: Aerobiology: proc. of the 3rd Internat. Symp. (Academic Press).

SMITH, C. U. M.: Molecular biology. (Faber).

STERZL, J. and RIBIA, H. eds.: Developmental aspects of antibody formation and structure. (Symp. Prague 1969). (Academic Press).

SYKES, M. K. and VICKERS, M. D.: Principles of measurement for anaesthetists. (Blackwell).

SYMINGTON, T.: Functional pathology of the human adrenal gland. (Livingstone).

THOMAS, J. H. and POWELL, D. E. B.: Blood disorders in the elderly. (Wright). Presented by John Wright & Sons Ltd.

TIETZ, N. W. ed.: Fundamentals of clinical chemistry. (Saunders).

TOSTESON, D. C. ed.: Molecular basis of membrane function. (Prentice-Hall).

TRINKAUS, J. P.: Cells into organs: the forces that shape the embryo. (Prentice-Hall).

TROUNCE, J. R. ed.: A guide to drugs in current use. (Med. & Tech. Publ.).

TUNNADINE, L. P. D.: Contraception and sexual life: a therapeutic approach. (Tavistock).

VAN FURTH, R. ed.: Mononuclear phagocytes. (Blackwell).

WADE, O. L.: Adverse reaction to drugs. (Heinemann).

WHITTOW, G. C.: Comparative physiology of thermoregulation. Vol. 1. (Academic Press).

WIED, G. L. and BAHR, G. F.: Introduction to quantitative cytochemistry, 2 vols. (Academic Press).

WILLIAMS, P. L. et al.: Basic human embryology. Ed. 2. (Pitman).

YOFFEY, J. M. and COURTICE, F. C.: Lymphatics, lymph and the lymphomyeloid complex. (Academic Press).

ZBOROWSKI, M.: People in Pain. (Jossey-Bass).

### News And Notes

### Burden Research Medal

The Burden Research Medal and Prize for this year have been awarded to Dr. J. Jancar, Consultant Psychiatrist at Stoke Park Hospital, Bristol. Dr. Jancar's article on Assessment Unit for the Mentally Retarded appeared in the April number of this Journal (p. 27).

### Examination Results, Degrees and Diplomas conferred

**F.R.C.P.**
J. F. Ackroyd
I. S. Bailey
D. R. Coles
R. D. G. Creery
R. S. Crow
M. S. Dunnill
P. C. Farrant
W. A. Gillespie
P. Jacobs
J. L. G. Thomson
G. Walters

**F.R.C.Path.**
N. F. W. Brueton
D. H. Johnson

**M.R.C.Path.**
T. F. Draisey
R. T. Wensley

### University of Bristol

**Ch.M.**
M. G. Smythe-Golby

**Ph.D. (Faculty of Medicine)**

F. J. Bourne

P. F. Flood

F. W. G. Hill

**D.P.H.**
Gay H. Alexander
N. Andeyaba
P. J. Blackburn
P. R. D. Boteju
K. J. M. Carruthers
J. Eskell
G-E. A. Farid
O. O. Kale
M. S. Khan
F. Kumagai
N. Onodera
A. E. Pointer
Jean Price
C. L. Samarasinghe
S. Stead
W. W. Williams.

**M.B., Ch.B.**
**With Honours:—**
R. M. Auty, M. Fouracres, Jane R. Savage, Katharine M. Walmesley.
**Pass:—**
K. Barnes, J. H. Baumer, S. P. Cembrowicz, P. A. Connell, M. H. Cullen, Anne Dixon, Josephine Emmerson, Josephine Forster, T. H. Frewin, J. B. F. Grant, R. M. Greenfield, P. J. Hardwick, A. R. Hearn, J. A. Heathcote, P. R. Hepburn, P. Hickling, M. C. Hime, D. J. Hobbs, N. R. J. Hooper, M. H. James, Julia M. Leach, D. A. Ley, R. J. Lichy, P. J. Magill, R. M. Mendelson, C. J. Molan, A. R. Mugridge, Sita M. Nath, J. R. Ogaba, Susan O'Neill, Gillian M. Osmond, Judith A. Palmer, J. S. Paynes, T. R. Penistan, T. J. Pocock, R. G. Potter, C. W. Richards, Ruth M. Rimmer, A. M. I. Sankies, R. W. Sawford, Jane E. Scull, Jessica Shipman, E. E. Smith, P. D. Stubbs, R. F. C. Swindells, M. A. Tettenborn, P. M. Trenchard, Carolyn M. Woodhouse, M. S. Rigler.

